# Optimizing NIAGADS sub‐cohorts based on APOE and age enhance Alzheimer's Disease gene discovery

**DOI:** 10.1002/alz70855_103974

**Published:** 2025-12-23

**Authors:** Kwanghyuk Danny Lee

**Affiliations:** ^1^ Baylor College of Medicine, Houston, TX, USA

## Abstract

**Background:**

AD is a devastating condition that affects millions in the US. To advance gene discovery and therapeutic development for AD, the Alzheimer's Disease Sequencing Project (ADSP) was launched as a presidential initiative in 2012. Since its inception, ADSP has collected over 80,000 sequencing samples through the National Institute on Aging Genetics of Alzheimer's Disease Data Storage Site (NIAGADS). While investigating the genetic factors underlying AD using NIAGADS, we observed significant heterogeneity in the phenotypes of sub‐cohorts, including sex, APOE genotypes, and age.

**Method:**

We hypothesized that this heterogeneity could hinder robust AD risk discoveries and aimed to generate a common dataset. We utilized the latest NIAGADS WES (R2) and WGS (R4) datasets, comprising 56,000 AD and healthy subjects. After quality control including the genetic ancestry prediction, we analyzed sub‐cohorts based on sex, age, age of onset, and APOE genotypes. Our pipelines were then applied to identify new AD candidate genes, which were subsequently validated through comprehensive computational and experimental methods to assess their performance.

**Result:**

We observed significant differences in APOE and age distributions between AD and healthy subjects. However, we also identified highly diverse age and APOE profiles across sub‐cohorts, leading us to pinpoint outlier sub‐cohorts. Applying our pipelines to the diverse R2 and R4 NIAGADS datasets, we found that removing these outliers improved AD gene discovery by enhancing replication of known AD genes (*p* 1.2e‐3 and 8.6e‐5 for R2 and R4, respectively) and increasing connectivity to known AD genes in the STRING network (*z* 5.62 and 5.98, respectively). Further analyses of extremely young and old AD cases within the remaining cohorts also facilitated better gene discoveries.

**Conclusion:**

The component cohorts of NIAGADS R2–R4 exhibit significant heterogeneity, as demonstrated by the diverse APOE and age distributions. Our approaches showed that optimizing these diverse cohorts based on APOE and age distributions improves AD gene recovery. The heterogeneity of AD cohorts also provides new opportunities to study smaller sub‐groups, such as age of onset and specific APOE profiles. These diverse NIAGADS cohorts could ultimately help define standard criteria for AD cases and control subjects, offering valuable insights for future research.

